# Host-, Environment-, or Human-Related Effects Drive Interspecies Interactions in an Animal Tuberculosis Multi-Host Community Depending on the Host and Season

**DOI:** 10.1155/2024/9779569

**Published:** 2024-06-10

**Authors:** Eduardo M. Ferreira, Mónica V. Cunha, Elsa L. Duarte, Renata Gonçalves, Tiago Pinto, António Mira, Sara M. Santos

**Affiliations:** ^1^ MED—Mediterranean Institute for Agriculture Environment and Development and CHANGE—Global Change and Sustainability Institute University of Évora Mitra, Évora7006-554Portugal; ^2^ IIFA—Institute for Advanced Studies and Research University of Évora Vimioso Palace, Évora7002-554Portugal; ^3^ Conservation Biology Lab Department of Biology University of Évora ÉvoraPortugal; ^4^ Centre for Ecology Evolution and Environmental Changes (cE3c) and CHANGE—Global Change and Sustainability Institute Faculty of Sciences University of Lisbon LisbonPortugal; ^5^ Biosystems and Integrative Sciences Institute (BioISI) Faculty of Sciences University of Lisbon LisbonPortugal; ^6^ Department of Veterinary Medicine University of Évora Mitra, Évora7006-554Portugal

## Abstract

In many Mediterranean ecosystems, animal tuberculosis (TB), caused by *Mycobacterium bovis*, is maintained by multi-host communities in which cattle and different wildlife species establish interaction networks contributing to *M. bovis* transmission and persistence. Most studies have addressed wildlife–cattle disease-relevant interactions, focusing on reservoir hosts, while disregarding the potential contribution of the so-called accidental hosts and/or neglecting wildlife–wildlife interactions. In this work, we aimed to characterise interspecies interactions in an endemic TB risk area and identify the ecological drivers of interaction patterns regardless of the pre-attributed role of host species on TB epidemiology. For that purpose, spatial–temporal indirect interactions between wildlife mammals and cattle, and between different wildlife species, were investigated through camera trapping. Second, five ecological hypotheses potentially driving species pair interactions in the wet and dry seasons were tested covering water and control sites: human presence (H1), landscape composition (H2), topography (H3), weather (H4), and natural food and water resources (H5). Wild boar (*Sus scrofa*), red deer (*Cervus elaphus*), and red fox (*Vulpes vulpes*) were the wildlife species mostly involved in indirect interactions. We found that indirect wildlife–cattle interactions were more frequent than wildlife interactions and, for certain species pairs, interaction rates were higher in the wet season in both wildlife–cattle and wildlife groups. Natural food and water resources (H5) was the most supported hypothesis that influenced the abundance of wildlife–cattle interactions, with positive effects during the dry season and negative effects during the wet season. In contrast, the abundance of indirect interactions between wildlife species was mainly supported by the human disturbance hypothesis (H1), with negative effects exerted on the dry season and variable effects on the wet season. Other tested hypotheses also influenced wildlife–cattle and wildlife–wildlife interactions, depending on the season and host species. These results highlight that indirect interactions, and thus conditions potentially favouring the transmission of *M. bovis* in shared environments, are determined by different ecological backgrounds.

## 1. Introduction

Wildlife–livestock interfaces are physical spaces where wildlife and domestic species can overlap in space and time, along with humans, and where they can potentially interact [1, 2]. Human activities (e.g., agricultural, husbandry practices, deforestation and industry) have been causing marked transformations in habitats (e.g., encroachment into natural areas, habitat fragmentation), shaping these interfaces [3, 4, 5]. With the loss of natural habitats due to anthropogenic land-use changes, many wildlife species are forced to live in close proximity to those interfaces. In addition, hunting activities have been leading to a notable overlap of large game hunting areas with cattle extensive farming in several regions [6, 7]. Such changes have profound effects on species interactions and thereby increase the risk of pathogen transmission and the (re)emergence of multi-host diseases [4, 8, 9].

Pathogens shared by wildlife and cattle that are of economic and public health concern are considered an increasing problem worldwide [10, 11, 12, 13]. In the last decades, various studies have been addressing wildlife–cattle interactions in the context of multi-host diseases, including animal tuberculosis (TB), covering different eco-epidemiological scenarios [14]. Animal TB is mainly caused by *Mycobacterium bovis* and is a globally distributed zoonosis, affecting cattle and a wide range of wild mammals [15, 16, 17, 18]. The negative economic impacts of TB on cattle are related to premature culling of animals, animal trade restrictions, rejections at slaughterhouses, and costly eradication plans when mandatory [19, 20]. Wildlife maintenance hosts, which vary across ecosystems, hamper eradication efforts via pathogen spilling back to cattle [17, 21, 22]. Transmission may not only occur when a susceptible host comes into close contact with an infected host (direct interaction: same location and time) but also when animals contact asynchronously through contaminated environments (indirect interaction: shared space use in different time frames) [15, 16, 23]. In this sense, defining these spatial–temporal interactions between mammal hosts is of major importance for understanding TB transmission [24, 25, 26]. This has been recognised as a critical step towards knowing where and when control actions should be prioritised [27, 28, 29].

Local and global studies have previously shown that direct interactions between wildlife hosts and cattle are scarce; in contrast, indirect interactions involving shared environments occur more frequently [14, 30, 31, 32]. Although explored in fewer studies, similar trends have been observed between different wildlife species, with indirect interactions being more frequent [33, 34]. Opportunities for indirect interactions among wildlife at the wildlife–cattle interface are of particular concern in systems where *M. bovis* circulates in multi-host communities along ecosystem boundaries, potentially favouring pathogen transmission [35, 36]. This is the case in Mediterranean ecosystems (Iberian Peninsula), where *M. bovis* is able to infect multiple domestic (cattle, pigs, and goats) and wildlife hosts (ungulates and carnivores) that occur in sympatry [37, 38, 39, 40].

In Mediterranean ecosystems, the availability and distribution of water and food resources are deemed important for animal aggregation and subsequent interspecies interactions [6, 41, 42], with summer–autumn periods promoting increased disease-relevant interactions [28, 41]. Some studies have examined the effect of host attributes (e.g., animal density; [6]), as well as of the environment and landscape contexts (e.g., land cover; [43]) on patterns of interactions between TB hosts at the wildlife–cattle interface. However, the relative importance of different ecological factors, and how they contribute to regulate interspecies interaction patterns in multi-host communities, remains poorly understood [44]. Moreover, multifaceted studies that also focus on non-reservoir hosts in the host–space–time axes and/or beyond the classic wildlife–cattle binomen are lacking. Considering accidental hosts and their interactions could help reconstruct missing links in *M. bovis* transmission chains, either among wildlife populations or from wildlife to cattle. Therefore, a community-based perspective when targeting complex multi-host TB systems is crucial [25, 36] to identify potential host species and to typify the group of animal interactions that most likely contribute to TB maintenance within the community [45].

In Portugal, red deer (*Cervus elaphus*) and wild boar (*Sus scrofa*) have been recognised as the most TB-relevant wildlife hosts, with reports of environmental contamination of natural substrates (soil and water bodies) in areas where wildlife TB is highly prevalent [46, 47, 48, 49, 50]. In this work, we aimed to increase global understanding of spatial–temporal indirect interaction patterns within a multi-host mammal community (cattle and wildlife: red deer, wild boar, red fox (*Vulpes vulpes*), and badger (*Meles meles*)), focusing on a high prevalence TB area within a Mediterranean agroforestry system of Southern Portugal.

Specifically, we aimed to:

- typify the interaction patterns between cattle and wildlife, and between wildlife species, and discuss these patterns in relation to pathogen transmission risk,
- compare the interaction rates between wildlife–cattle and between wildlife groups in the dry and wet seasons, and
- evaluate the potential effect of a set of 18 ecological factors related to human disturbance, landscape composition, topography, weather, and natural resources on both wildlife–cattle and wildlife–wildlife interactions in the dry and wet seasons.

## 2. Materials and Methods

### 2.1. Study Area

**Figure 1:**
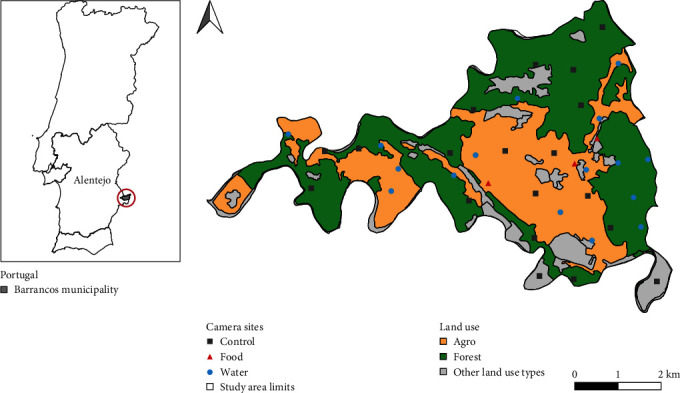
Study area location in Barrancos region, Portugal, showing camera sites and main land uses.

This study was conducted in Barrancos, located in Southeast Portugal (Alentejo region), close to the Spanish border (38˚08′N; 6˚59′W) (Figure 1). This area is considered a hotspot for TB in cattle and is included in the official epidemiological TB risk area where special measures (a mandatory veterinary examination of carcasses to search for TB-compatible lesions) apply to hunted big game species (red deer and wild boar) [48, 51, 52]. Ungulates are abundant in the region (wild boar density = 3–4 individuals/km^2^; red deer density = 4–8 individuals/km^2^) [53]. Barrancos is an important *Montado* region (i.e., woodland, a savannah-like open tree forest) with extensively cattle breeding in sympatry with wildlife (e.g., big game). Herd TB prevalence was estimated at 1.83% for the Alentejo region in 2022 [54]. A local study specifically conducted in Barrancos in 2014–2015 points towards a TB prevalence of 3.1% and 1.8% for red deer and wild boar, respectively [55]. While official numbers are remarkably lower, a meta-regression and systematic review analyses estimated the pooled TB prevalence at a national scale as 27.5% and 13.3% for the red deer and wild boar, respectively [56].

The study area (SA) has a Mediterranean climate, with mild and wet winters and hot and dry summers. Mean annual temperature ranges from 5 to 14°C during the winter (January), and from 15 to 34°C during the summer (July) (Beja; 1981–2010; [57]). During this study period, the mean temperature in January was 8.9°C and in July was 25.5°C. The average annual precipitation is 555 mm, concentrated between October and May. The topography is characterised by gentle to moderate undulating relief, with altitude ranging between 160 and 350 ma.s.l. The landscape is dominated by holm oak (*Quercus rotundifolia*) *Montado*, with varying tree and shrub density (Agro: holm oak stands with low or absent shrub cover due to grazing and other pastoral activities; Forest: holm oak stands or mixed woodland patches with high shrub cover) (Figure 1). Other less representative land cover types include olive groves and few shrub and agricultural area mosaics.

### 2.2. Study Design

We used camera-trapping to assess spatial–temporal patterns of interactions involving wildlife–cattle and wildlife–wildlife species over a year (from April 2021 to April 2022). Besides cattle, we used as target species the TB reservoir hosts described for Portugal (red deer and wild boar [17, 53]), and two other susceptible species that occur in the region: the red fox and the badger [38, 58].

We selected five free-ranging adjoining farms with similar management practices, comprising an area of ~3,048 ha (farm size ranging from 148 to 980 ha), with an average of 136 adult cows per farm. A 1 km grid was overlaid on the SA [59, 60]. One camera was installed on each 1 km^2^ cell, to assure spatial independence of sampling sites and land cover representativeness. From this grid, we first selected key sites (water and supplementary food sites; [24])—known as important aggregation points between species—prioritising sites located in different grid cells [61], and an even distribution across farms. The remaining empty cells were defined as control sampling sites, and camera-traps were placed on their centroids. A total of 38 sampling sites (hereafter called camera sites; Figure 1) were defined: three food sites for cattle (hay feeders); 16 water sites (natural water sources and water trough) and 19 control sites (without any water sources or supplementary food, e.g., forest animal path). Minimum distance between camera sites averaged 686 m (range: 350–1,300 m).

Each camera site consisted of a single camera-trap (Busnhell Trophy Cam HD Aggressor or Reconyx Hyperfire) placed 30–50 cm above the ground, attached to trees or artificial stakes. At water and food sites, the cameras were facing towards areas highly used by cattle and wildlife to maximise the detection of interaction between different species. At control sites, we prioritised animal trails or other areas (e.g., resting sites) potentially used by cattle and wildlife in suitable habitats. No bait of any kind was used. We programmed cameras to operate 24 hr a day, taking three sequential pictures per trigger with a 30-s delay between consecutive triggers [24, 61]. On average, every 10–15 days, we visited camera sites for battery and memory card replacement.

### 2.3. Data Coding and Processing

Pictures recorded by each camera were individually classified by visual observation. The following information was recorded in an Excel database: camera coordinates, camera site type (water, food, or control), target species (cattle, red deer, wild boar, red fox, and badger), and number of individuals (minimum number of individuals recorded in each picture). In addition, date–time of picture capture were retrieved using the open-source software ExifTool [62]. An independent observation of the same species (hereafter called “detections”) was considered at a given camera site when pictures were taken at least 15 min apart [6, 24, 63].

If cattle were unable to reach a given camera site in a certain period (due to cattle grazing rotation and management), that period from that camera was excluded from analyses. We assumed that the fences were permeable to wildlife [6], as confirmed in the field and several times in the camera pictures. The three camera sites initially classified as food sites had no food for long periods of time, and thus were excluded from further analyses.

### 2.4. Definition and Estimation of Interactions

An indirect interaction was defined as the detection of one species at a given camera site, following the detection of another species within a pre-established critical time window, CTW, related to estimated *M. bovis'*s environmental survival time. A CTW of 3 days for the dry season (June–September) and of 12 days for the wet season (October–May) was assumed, following the procedures of Kukielka et al. [24] and Cowie et al. [33], applied in a similar eco-environmental context (Figures S1 and S2). A direct interaction was defined whenever individuals of different species were captured in the same picture [14], although it was not analysed in this study due to the much lower number of observations recorded.

The number of indirect interactions was calculated for each camera site and month, discriminated by species pairs. The species pairs considered in this study are composed of the combinations of the five target species and are divided into two groups: the wildlife–cattle group includes four species pairs: BT_CE (cattle—red deer), BT_SS (cattle—wild boar), BT_MM (cattle—badger), and BT_VV (cattle—red fox); and the wildlife group includes six species pairs: CE_MM (red deer—badger), CE_SS (red deer—wild boar), CE_VV (red deer—red fox), SS_MM (wild boar—badger), VV_MM (red fox—badger), and VV_SS (red fox—wild boar). For each species pair and camera site, we calculated monthly rates of indirect interactions (*RatesInt*) as a function of the number of interactions (nr of interactions) per time (*RatesInt* = nr of interactions/time), adapted from Ferreira et al. [14] study. Time was expressed as a proportion, corresponding to active camera days (days when cameras were operational and recording without any interference) divided by the number of days in a given month. We summarised *RatesInt* by species pairs and seasons (indirect interactions/month/camera), computing *RatesInt* means along with the corresponding standard errors. Generalised linear models (GLM) were used to inspect potential differences in *RatesInt* between wildlife–cattle and wildlife groups, across seasons.

### 2.5. Human, Landscape, and Environment Predictors

To address the third objective, we defined a total of 18 eco-environmental predictors that influence the abundance of the target species and thus may influence species interaction patterns. These predictors were arranged according to five ecological hypotheses that might regulate species interactions: (H1) human disturbance (*n* = 4 predictor variables); (H2) landscape composition (*n* = 5); (H3) topography (*n* = 3); (H4) weather (*n* = 3); and (H5) natural food and water resources (*n* = 3) (Table 1).

We estimated human disturbance (H1) for each camera site through the total number of days with human records (visually extracted from pictures); and through the Euclidean distance of camera sites to the nearest houses, to hunting sites (stand sites for hunting, where baiting is placed nearby for attracting wildlife) and road density metrics of unpaved roads (length of roads/total area within a given neighbourhood) in the SA (Quantum GIS v. 3.0.3; [69]). For landscape composition-related predictors (H2), we computed the proportion of land cover, considering the main land uses (agroforest and forest) occurring in the SA; the Shannon landscape diversity index, and the Euclidean distance of camera sites to forest edges. Those metrics were obtained from the Corine Land Cover (2018) dataset (European Union, Copernicus Land Monitoring Service, European Environment Agency) and were retrieved from the “landscapemetrics” R package [70]. In addition, tree cover density was derived from the Tree Cover Density (2018) dataset (Copernicus Land Monitoring Service, European Environment Agency) (Table 1).

Regarding topographic predictors (H3), we estimated elevation from a 30-m digital elevation model (DEM) and derived terrain ruggedness index and slope from the DEM using Quantum GIS v. 3.0.3. Weather-based predictors (H4; i.e., Rain and Temp) were obtained from data collected at a local weather station. Lastly, for H5, the water content (Water_cont) was visually estimated based on the area covered by standing water (using some marks in situ to retrieve estimates) during field work visits throughout the sampling period. The typology of each camera site—Station_site (control or water)—was used as a categorical variable. The normalised difference vegetation index (NDVI) was derived from the LANDSAT 8 image collection (level 2, Tier 1), with a 30 m spatial resolution, and processed in Google Earth Engine [71]. The NDVI has shown a high correlation with vegetation biomass and dynamics in various ecosystems worldwide. Several authors have used NDVI to assess vegetation productivity—representing resource quantity and quality—and the dynamics of habitat use by wild mammals, including ungulates and carnivores [72, 73, 74]. For this reason, we used NDVI as a proxy for natural food availability. We only retained high-quality images with ≤5% of cloud cover considering the whole SA (more details are available in [75]). For the missing data in our time series (a 3-month gap, non-consecutive months), we used images from the month before and after (time interpolation; [76]) to estimate the NDVI values [77].

A multi-scale approach was carried out to cover a wide range of scales and thus maximise potential responses with the target species [78]. Continuous predictors not based on distances (Dens_roads, TreeD, Altitude, Rugg, Slope, and NDVI) were stacked in a 30 m spatial resolution multi-raster layer. We then applied the following spatial scales of analysis: 90, 240, and 510 m focal-radius moving window as a proxy for 100, 250, and 500 m neighbourhood scales of analysis around camera sites. Mean was used to summarise the raster values within each spatial scale A similar procedure (in terms of scales) was applied to Agro, Forest, and Shidi using a spatial resolution basis of 10 m, and thus a focal-radius moving window of 100, 250, and 500 m (Table 1).

We also estimated the relative abundance index of each target species (e.g., RAI), discriminated by camera site and season, to be used as a proxy of animal density in the modelling process. Animal abundance was calculated as the number of detections of each species in a month/(number of active camera days/number of days of a given month).

### 2.6. Modelling: Hypotheses Explaining Interspecies Indirect Interactions

Interaction analyses were conducted separately for each species pair, and for the dry and wet seasons, allowing the identification of potential differences in the effects of predictors driving interactions between seasons. As pre-modelling procedures, we checked for outliers and inspected collinearity among variable predictors. Pairwise Spearman correlations were calculated among all predictors to check for multicollinearity. Numeric predictors with skewed distributions were transformed (square-root, logarithmic, or arcsine) to approach normality and to reduce the influence of extreme values [79]. In addition, all continuous predictors were standardised, allowing comparisons of their strength in the modelling process.

We fitted the response variable—number of species interactions—to generalised linear mixed models (GLMM) with a Poisson or negative binomial family distribution and log link (package “glmmTMB” [80]), using camera site as a random factor because each camera site was sampled repeatedly through time. The log of the number of active camera-days was used as offset in the models to integrate sampling effort between camera sites over time [24]. This procedure avoided transforming count data (log-transformed data or *RatesInt*), as recommended by Zuur et al. [79] and O'Hara and Kotze [81].

The five ecological hypotheses (H1–H5) were independently evaluated [82], first through simple models, testing one predictor at a time. These simple models always included the abundances of each species (RAI) involved in a given species pair interaction as fixed predictors, since higher host abundance increases interaction levels [14]. Then, if more than one predictor was informative within a hypothesis, a multivariate model was built for each hypothesis with all informative predictors.

 
*Model example: species pair AB|season*

Number of interactions~*animal abundance* (*A*) + *animal abundance* (*B*) + predictor *X* + *random* (1|camera site), *offset* (log (camera days)), *family* (Poisson/negative binomial).

A predictor variable was considered informative when: (1) the 95% confidence intervals (CI: 95%) of the variable coefficient being tested did not include zero; and (2) a deltaAICc > 2 (*Δ*AICc; Akaike's information criterion adjusted for small sample sizes) was obtained when comparing the tested model with the reference model (without the specific predictor; [79, 83, 84]). If highly correlated informative predictors (*r* > 0.7) were identified, we only retained the one producing a lower AICc to be included in the multivariate model. This procedure also involved comparing multiple scales for a given predictor. Multivariate models were built with all possible combinations of the informative predictors of each hypothesis, always keeping animal abundance (RAI) in all competing models, and limiting each model to a maximum of four predictors to avoid model instability. We selected the best multivariate model for each hypothesis using AICc. Models having a *Δ*AICc < 2 are considered equally supported. When several models had *∆*AICc < 2 : 1) all associated predictors were included in a single best multi-model [85] if ≤ four predictors were selected; (2) all models within *∆*AICc < 2 of the top-ranked models were retained for interpretation, otherwise.

The dredge function (R package “MuMIn” [86]) was used for model selection. Once we identified all the best models for the hypotheses tested (H1–H5), we again ran the models with a restricted maximum likelihood (REML). Since it is important to assess model adequacy [87, 88], models were evaluated and validated using diagnostic tools (normality, outliers, and zero inflation) available in the “DHARMa” package [89].

## 3. Results

We obtained a total of 15,537 detections of cattle and target wild mammal species over 6,170 effective trap days across the 35 camera sites (mean = 176 ± 61 sd trap days per camera site) during the study period. Cattle were the most frequently detected species (66.8%; *n* = 10,379). Red fox (10.5%; *n* = 1,631), red deer (8.6%; *n* = 1,335), and wild boar (7.3%; *n* = 1,141) were detected in similar numbers and were widespread in the SA (detection in >85% of camera sites). The badger occurred at lower rates (2.5%; *n* = 382), although it was also widespread in the SA (detection in >75% of camera sites).

### 3.1. Wildlife–Cattle and Wildlife Species Interactions

**Figure 2:**
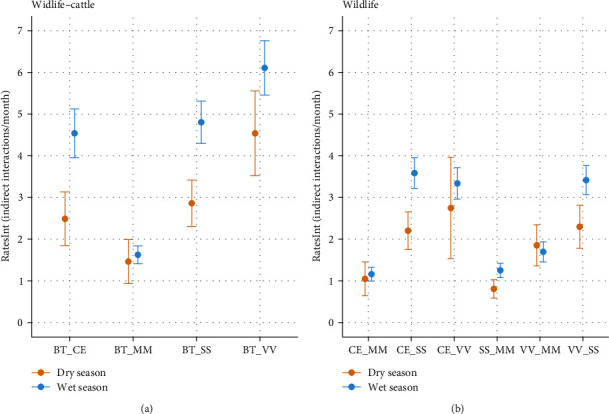
Weighted means and standard errors of *RatesInt* (indirect interactions/month) summarised by species pairs and seasons and displayed by animal group ((a) wildlife–cattle; (b) wildlife). Species pair acronyms are (BT_CE) cattle—red deer; (BT_MM) cattle—badger; (BT_SS) cattle—wild boar; (BT_VV) cattle—red fox; (CE_MM) red deer—badger; (CE_SS) red deer—wild boar; (CE_VV) red deer—red fox; (SS_MM) wild boar—badger; (VV_MM) red fox—badger; and (VV_SS) red fox–wild boar.

Wildlife–cattle indirect interactions represented 52.7% (*n* = 3,619) of the interaction data (only 0.1% (*n* = 7) were direct interactions involving cattle). The wildlife species that were most frequently involved in these interactions were the red fox (BT_VV; mean *RatesInt*: wet season = 6.1 and mean *RatesInt*: dry season = 4.5), followed by the wild boar (BT_SS; mean *RatesInt*: wet season = 4.8 and mean *RatesInt*: dry season = 2.8) and red deer (BT_CE; mean *RatesInt*: wet season = 4.5 and mean *RatesInt*: dry season = 2.5). The badger (BT_MM; mean *RatesInt*: wet season = 1.6 and mean *RatesInt*: dry season = 1.5) interacted less frequently with cattle (Figure 2(a)). Interactions with cattle involving the three most detected species (red fox, wild boar, and red deer) occurred in all farms, at more than 80% of camera sites during the wet season, and at 30%–60% of camera sites in the dry season. Interaction rates were significantly higher in the wet season for the pairs BT_VV (GLM; coef: wet season = 0.361, CI: 95% (0.050; 0.672)), BT_SS (GLM; coef: wet season = 0.304, CI: 95% (0.024; 0.585)), and BT_CE (GLM; coef: wet season = 0.441, CI: 95% (0.167; 0.714)).

Indirect interactions between wildlife represented 46.8% (*n* = 3,210) of the interaction data (only 0.4% (*n* = 25) were direct interactions). The wildlife species pairs most frequently interacting were CE_SS (mean *RatesInt*: wet season = 3.6 and mean *RatesInt*: dry season = 2.2), CE_VV (mean *RatesInt*: wet season = 3.3 and mean *RatesInt*: dry season = 2.7), and VV_SS (mean *RatesInt*: wet season = 3.4 and mean *RatesInt*: dry season = 2.3) (Figure 2(b)). Indirect interactions between the three main species (red fox, wild boar, and red deer) occurred at more than 80% of camera sites during the wet season, and at 40%–60% of camera sites during the dry season. Interaction rates were significantly higher in the wet season for the pairs CE_SS (GLM; coef: wet season = 0.283, CI: 95% (0.031; 0.535)), CE_VV (GLM; coef: wet season = 0.302, CI: 95% (0.045; 0.559)), and VV_SS (GLM; coef: wet season = 0.297, CI: 95% (0.038; 0.556)).

### 3.2. *RatesInt* between Wildlife–Cattle and Wildlife Groups

Globally, interaction rates (*RatesInt*) were higher in the wet season for both wildlife–cattle and wildlife groups when compared to the dry season. The mean interaction rates of the wildlife–cattle group were 1.8 and 1.6 times significantly higher than the wildlife rates for the dry and wet seasons, respectively (GLM dry season; coef wildlife: −0.156, CI: 95% (−0.285; −0.0269); GLM wet season; coef wildlife: −0.269, CI: 95% (−0.354; −0.184)).

### 3.3. Ecological Hypotheses Driving Species Interactions

**Figure 3:**
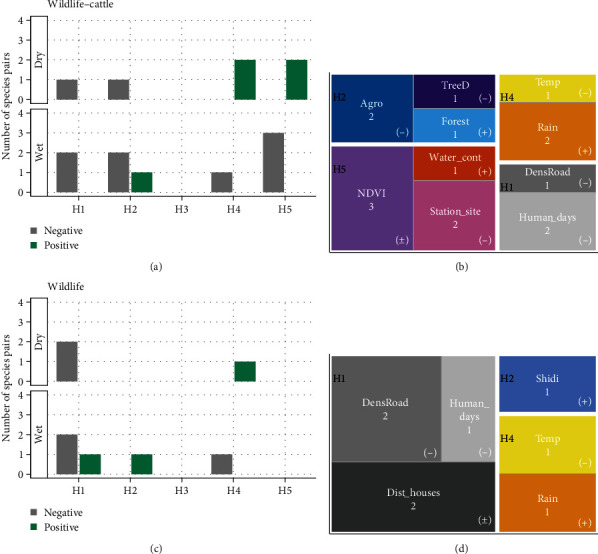
Number of species pairs influenced by ecological hypotheses regarding indirect interactions, displayed by wildlife–cattle (a) and wildlife (c) groups and considering sampled seasons. For each hypothesis, the sign of the coefficient effect is shown (positive, negative, or null). Treemaps show the number of times the tested predictors, underlying ecological hypotheses, were associated with species pair interactions, displayed by wildlife–cattle (b) and wildlife (d) groups.

All models were fitted with a Poisson family distribution. The predictors Slope, Rugg, Agro, and Forest were not used simultaneously in the same model due to multicollinearity problems. Locations with high terrain ruggedness had also higher slope (rs = 0.99) and low percentage of Agro (rs = −0.73). On the other hand, locations with high percentage of Agro had low percentage of Forest (rs = −0.74). Model residual patterns revealed a good to adequate fit of most of the models to the data (Figures S3, S4, S5, and S6: DHARMa diagnostic plots showing residual, dispersion, and zero-inflation fits of the tested models). Four of the five ecological hypotheses tested were significantly associated with abundance of wildlife–cattle interactions, covering one to three species pairs, depending on the hypothesis (Table 2 and Figure 3(a)). Three of the five ecological hypotheses tested were significantly associated with the abundance of wildlife interactions, covering from one to four species pairs, depending on the hypothesis (Table 3 and Figure 3(c)). Wildlife–cattle interactions were most related to natural food and water resources hypothesis (H5) (Figure 3(b)), while wildlife interactions were often associated with human disturbance hypothesis (H1) (Figure 3(d)).

#### 3.3.1. Modelling: Wildlife–Cattle Interactions

The number of wildlife–cattle interactions, involving the red fox and wild boar, increased in areas with a lower human presence during the wet season (H1, models: BTVV_wH1 and BTSS_wH1; Table 2). Additionally, in this season, interactions encompassing the red deer, wild boar, and badger increased in more forested areas (e.g., areas with low Agro cover; H2, models: BTCE_wH2, BTSS_wH2, and BTMM_wH2). More interactions between cattle and red deer were associated with low-temperature periods (H4, model: BTCE_wH4). The higher abundance of interactions, covering red deer, red fox, and wild boar, occurred in areas where natural resources are less abundant (i.e., control sites and less productive areas (NDVI)) (H5, models: BTCE_wH5, BTVV_wH5 and BTSS_wH5). During the dry season, wildlife–cattle interactions increased in areas with lower road densities, as evidenced for the red fox (H1, model: BTVV_dH1), and in areas with lower tree cover, in the case of the red deer (H4, model: BTCE_dH2). Rain had a positive influence on the abundance of wildlife–cattle interactions (H4, models: BTCE_dH4 and BTVV_dH4), and interactions were more frequent in sites with higher water content and in more productive areas, for carnivores and ungulates, such as the red fox and the red deer, respectively (H5, models: BTVV_dH5 and BTCE_dH5). Overall, animal abundance had a strong effect size in all models: with one-point increase in animal abundance (wildlife or cattle), number of interactions would be expected to increase by an average IRR of 2.93 (sd = 0.58), holding all variables constant. Ecological predictors, linked to the study hypotheses, had a lesser pronounced effect (positive predictors: average IRR = 1.49, sd = 0.46; negative predictors: average IRR = 0.79, sd = 0.15).

#### 3.3.2. Modelling: Wildlife–Wildlife Interactions

During the wet season, wildlife interactions involving ungulates increased at longer distances to houses (H1, model: CESS_wH1; Table 3), and in areas with lower road densities, for the species pair CE_VV (H1, model: CEVV_wH1). Human disturbance, through human presence, also had a negative effect on the abundance of wildlife interactions in this season: in this case between wild boar and red fox (H1, model: VVSS_wH1). Furthermore, wildlife interactions—encompassing VV_SS and CE_SS species pairs—increased in areas with higher landscape diversity (H2, model: VVSS_wH2) and when the temperature was lower (H4, model: CESS_wH4). In the dry season, wildlife interactions also increased as a function of low road densities, specifically for the SS_MM species pair (H1, model: SSMM_dH1), while interactions between the red fox and wild boar increased at reduced distances from houses (H1, model: VVSS_dH1). Furthermore, wildlife interactions—involving badger and red deer—increased in rainy periods (H4, model: CEMM_dH4). Overall, with a one-point increase in animal abundance (wildlife), the number of interactions would be expected to increase by an average IRR of 2.52 (sd = 0.39), holding all variables constant. Ecological predictors, linked to the study hypotheses, had a lesser pronounced effect size. Positive predictors had an average IRR of 1.26 (sd = 0.27), while predicators exhibiting a negative relation with the number of wildlife interactions had an average IRR of 0.80 (sd = 0.149), meaning that a one-point increase in a given predictor would be expected to result in a decrease in the rate ratio for the number of interactions.

## 4. Discussion

Pathogen transmission at shared interfaces is a heterogeneous and dynamic process, significantly dependent on spatial and temporal processes. Despite being overlooked in certain TB risk areas, characterising spatial–temporal variation in interaction patterns, addressing all relevant hosts, is essential to properly understand pathogen transmission dynamics in complex animal communities.

We demonstrated that (1) wildlife–cattle and wildlife indirect interactions occur frequently. All the target species contributed to the network of disease-relevant interactions yet, wild boar, red deer, and red fox were the wildlife hosts mostly involved in indirect interactions across seasons. Regardless of the group considered, species pair interactions were generally higher in the wet season; (2) the rates of indirect interaction involving wildlife–cattle were higher than the interactions between wildlife species, in both seasons; (3) several hypotheses influenced indirect interactions, although responses differed among groups and seasons. Wildlife–cattle interactions were more frequently related to the natural food and water hypothesis (H5), while wildlife indirect interactions were more associated with the human disturbance hypothesis (H1).

### 4.1. Wildlife–Cattle and Wildlife Interaction Patterns

Interspecies direct interactions were rare, as previously documented in other studies [31, 33, 63]. This highlights that even generalist species, with similar ecological requirements, tend to partition resource use and habitat exploitation spatially and temporally [59, 60, 90]. On the other hand, wildlife–cattle and wildlife indirect interactions were frequent and widespread throughout the study area. Such results are consistent with previous findings reported in Mediterranean ecosystems, supporting the hypothesis that *M. bovis* transmission (and other multi-host pathogens with similar excretion routes) is mainly indirect through contaminated shared environments [23, 36, 91]. Agroforestry systems like *Montado—*known as *Dehesa* in Spain—are highly complex structures often considered as high nature value farming systems, supporting high levels of biodiversity [92]. Human activities (e.g., hunting interests), along with other ecological and social factors, have been shaping these interfaces, promoting a notable overlap between wildlife (e.g., big game hunting) and cattle farming. Consequently, *Montado* interfaces have become increasingly interconnected, requiring improved management practices, as shared space is expected to favour interspecies disease transmission. Indeed, the long-term excretion and viability maintenance of *Mycobacterium tuberculosis complex* bacteria (MTBC) in environmental substrates [50] increase animal exposure risk, particularly in animal aggregation areas that are asynchronously used by different species. In Mediterranean Spain, host species richness has been correlated with increased community competence to maintain and transmit MTBC, oppositely to other epidemiological settings where biodiversity could favour a “dilution effect” and moderate pathogen transmission [93].

Wild boar, red deer, and red fox were the wildlife hosts more frequently involved in indirect interactions, as shown in previous studies conducted in similar environments [6, 91, 94, 95]. The positive relationship between wildlife/cattle abundance and the number of interactions is notable, with significant effects observed in all tested models. This pattern is compatible with a density-dependent mechanism, a hypothesis previously suggested in the context of animal interactions within disease systems [96], including TB [6, 14]. Thus, higher interaction events involving ungulates and red foxes are expected, as they are more abundant in our study area. On the other hand, the low number of indirect interactions involving badgers could be related to their lower local abundance, in contrast to other Iberian environments (e.g., Asturias, Northern Spain) and other European TB contexts (e.g., UK), known to have higher badger population densities and where significant shared space between badgers, cattle, and other wild mammals has been documented [91, 97]. From an epidemiological perspective, these results highlight that reservoir hosts (wild boar and red deer) potentially play a key role in disease transmission in the study region and should therefore receive increased attention [53]. Wild boar has been identified as a TB maintenance host in most study sites across the Iberian Peninsula. In the context of multi-pathogen networks (study conducted in Spain), wild boar is considered as the key and most connected species of the system community, bridging several hosts relevant to the epidemiology of MTBC [45, 53]. Also, TB prevalence in wild boar and the red deer was considered an important factor positively linked to TB in cattle farms of Iberian regions [98, 99]. Nevertheless, additional research (e.g., pathogen excretion patterns and burden) is needed, including for other non-reservoir hosts, given their potential to indirectly interact with various species, as the case of the red fox in our study. The red fox is a generalist carnivore that can exploit a variety of habitats, including farm-related sites [100], and was recognised as a spillover host in certain regions [101]. However, despite recent insights about MTBC environmental contamination in the Iberian Peninsula [50, 102], the relative importance of certain TB hosts—including the red fox—to environmental contamination remains poorly understood in TB risk areas.

The higher rates of interactions during the wet season may be due to different factors (e.g., species-specific behaviours, animal density; [6, 28]) but are mostly driven by two. First, the higher availability and abundance of resources during the wet season (e.g., autumn). While summer periods tend to drive species aggregation around spatially limited resources (e.g., water sites), the wet season is characterised by high availability and abundance of natural food and water sites. This could attract species to new areas, resulting in indirect shared space across landscapes, which can be significant when considering common and generalist species as in the case of red deer, wild boar, and red fox. Second, in our study area, cattle are confined to fewer grazing plots during the dry season when compared to the wet season. This may also be a plausible explanation for the lower rates of interactions involving cattle in the dry season (less sites where animal hosts may engage), and contradicts other studies that referred to a generalised increase in indirect interactions in dry periods (but also in autumn periods) [24, 33].

### 4.2. Differences between Wildlife–Cattle and Wildlife Interaction Rates

Wildlife–cattle indirect interaction rates were almost two times higher than wildlife interaction rates in both seasons. Triguero-Ocaña et al. [95] have also found that wildlife–cattle interactions involving red deer, fallow deer (*Dama dama*), and wild boar were more frequent than interactions between wildlife species. Such patterns could be related to how species partition resources across the landscape and to species-specific behaviour traits, which may differ between the two groups. The response of wildlife to cattle presence (e.g., behavioural effects) can be heterogeneous when considering different animal species and landscape contexts [103]. Although some studies have shown that cattle presence had a negative influence on space use by some carnivore host species (e.g., badger and red fox; [64, 104, 105]), others have shown that cattle presence was positively associated with wildlife occurrence, namely for the wild boar and red deer in agroforestry areas [6, 63]. Regarding the spatial–temporal profiles of wildlife species, some studies demonstrated that even habitat-generalist carnivores (e.g., red fox and badger) may exhibit contrasting habitat preferences at a small-scale in agroforestry systems [59]; and mesocarnivore co-occurrence is limited by landscape homogeneity [67], a trait observed to some extent in our study area. In addition, species (e.g., ungulates) can segregate in terms of space and time to avoid competitive and agonistic encounters [60]. Therefore, in Mediterranean ecosystems characterized by multifunctional landscapes, interspecies avoidance through shared resources between cattle and wildlife should be smaller [106] than between nocturnal wildlife species with more similar activity rhythm periods, sizes, and diets [94, 107]. In turn, animal co-occurrence patterns may dictate indirect interaction between hosts through shared environments, and thus having considerable influence on animal TB epidemiology.

### 4.3. Ecological Hypotheses Driving Wildlife–Cattle and Wildlife Indirect Interactions

The abundance of natural food and water resources (H5) markedly influenced wildlife–cattle indirect interaction patterns, particularly those involving red deer, wild boar, and red fox. Our results indicate that, in the wet season, wildlife–cattle interactions increased in less productive areas (e.g., forested areas with high shrub cover), and around control sites; while during the dry season, wildlife–cattle indirect interactions were associated with more productive areas and occured significantly more at sites with higher water content. Water and food resources (natural and artificial) have been previously identified as key components, highly used by both cattle and wildlife at shared interfaces, thereby favouring interspecies transmission of *M. bovis* [24, 31, 32]. The seasonal patterns evidenced in our work may be related to changes in resource availability and abundance throughout the year. In the wet season (mainly autumn and early winter), acorns (important for ungulates) and pastures (important for cattle, ungulates, and carnivores) are abundant in the study area and more water sites are available. Oppositely, water and natural food resources tend to be scarce and more spatially limited in the dry season. Given that, in the wet season, although lower levels of wildlife–cattle interactions are expected at specific sites (due to the use of different resources), spatial co-occurrence between cattle and wildlife continues to take place outside key resource areas in different habitats, as documented in other studies [91]. On the other hand, highly productive natural food areas and water sites become more attractive to numerous animal hosts in the dry season. This leads to spatial aggregation of hosts at specific sites, increasing the probability of indirect interactions around key resources, as shown in previous studies [31, 41].

The tested hypotheses also revealed that the wildlife–cattle interactions increased in areas with low human presence (H1), more dense vegetation (H2; e.g., Forest), and in periods of low temperature (H4) during the wet season; and, during the dry season, wildlife–cattle interactions increased in areas with lower road densities (H1), in more open areas (H2; i.e., less tree cover) and during rainy periods (H4). The effect of land use [41] and human disturbance (e.g., hunting effects; [63]) on species interactions have previously been suggested in other Mediterranean areas. In addition, weather effects (H4) can also play a role in interactions involving cattle, since wildlife movement behaviour on farms can be affected by temperature and rain (e.g., red fox and badger; [108]). Overall, our results indicate that the critical conditions for animal interactions, depending on the season, are shaped by several ecological components. This highlights the importance to consider a broad range of different ecological factors when determining *when* and *where* disease transmission can occur.

Effects associated with human disturbance hypothesis (H1) were observed for wildlife interactions as well, which have been largely understudied in the context of TB until now. During the wet season, wildlife interactions were negatively related to road density and human presence, and positively related to the distance to houses. In the dry season, lower road densities and increased distances from houses were also found to be key conditions where transmission of *M. bovis* may be favoured between wildlife species (i.e., high rates of indirect interactions). Studies have demonstrated that wildlife occurrence is strongly affected by different anthropogenic factors, such as roads (e.g., ungulates and carnivores in relation to dirt roads; [109, 110]), human presence (e.g., ungulates; [66]), or even human settlements (e.g., carnivores; [111]). We hypothesised that in the study area, wildlife species (both carnivores and ungulates) tend to avoid unpaved roads—they are frequently used by local workers and hunters throughout seasons—and areas close to houses (particularly interactions involving the red deer). By adopting such behaviours, species reduce the probability of disturbance, which, as expected, results in lower abundance of indirect interactions through common space use in those areas. In the dry season, the higher probability of wildlife interactions in areas close to houses could be explained by the characteristic behaviour of the species involved, namely the red fox and wild boar. These are opportunistic species that can take advantage of resources close to human settlements when those resources are scarce elsewhere, as documented in other Mediterranean areas and habitats [64]. This may also explain why wildlife indirect interactions involving those species increase in more heterogeneous areas (H2), but in this case, evidenced during the wet season when various resources are often available across different habitats. Finally, models showed that wildlife interactions were influenced by weather conditions (H4; ungulates in relation with temperature and red deer and badger in relation to rain). We hypothesised that during the wet season, species home range could increase as a function of temperature, as documented for ungulates and some carnivores [68, 112]. As a result, this can lead species to use different spatial resources, likely reducing the abundance of interactions under these circumstances. On the other hand, species can boost their activity during the dry season in rainy periods (very infrequent events), which could be linked to increased prey activity and/or immediate water availability, for instance. Because resources are more limited in the dry season, such patterns can result in negligible spatial segregation, and thus probably increase indirect interactions between wildlife species, particularly at specific resource sites (e.g., water sites).

Overall, improving our understanding of the ecological and environmental drivers underlying disease-relevant interactions at the wildlife–cattle interfaces is likely to provide valuable insights into the real nature of pathogen transmission events. This knowledge can help refine and guide effective control actions in risk areas wherein disease still persists. Currently, TB surveillance in wildlife in Portugal almost exclusively relies in veterinary inspection of hunted large game animals in specific areas with endemic circulation of *M. bovis*. Moreover, conventional biosecurity measures can be particularly difficult to implement in animal extensive production systems, posing a considerable challenge for controlling multi-species pathogens. Still, additional preventive measures could be considered for disrupting *M. bovis* transmission chains. One example could involve implementing selective fencing and gating systems in specific areas where wildlife and cattle frequently share space, and where increasing interaction rates are expected (e.g., water sites in the dry season; [113]). Data from the present study may guide future actions as it could help refine disease risk maps, which presently mainly rely on data from disease breakdowns in cattle herds. Furthermore, wildlife densities—given their role in our study—should be closely monitored, along with environmental sampling to assess contamination of natural substrates, particularly in areas highly used by different hosts.

### 4.4. Study Limitations and Future Perspectives

We identified three main aspects that should be further scrutinised by researchers in the multi-host TB context: first, in our study, the even distribution of cameras across the landscape, encompassing different land uses, enhances the representation of features influencing animal detection proportionally to their availability. However, this does not eliminate the overall detection bias arising from the landscape, which can influence the field of vision of camera traps (e.g., reduced detection field in dense environments compared to open areas). Future studies on interaction patterns should integrate new tools (e.g., occupancy models) to address imperfect detection of individuals. Additionally, exploring animal-based metrics (e.g., via REM—random encounter model) that consider the collective viewsheds of a camera array could improve animal detection rates and related estimates (e.g., interactions) across varying spatial gradients and external drivers [59, 114]; second, host behaviour may determine the relative importance of a host within a disease system. Even if not very abundant, the behavioural repertoire could favour an increased contact with other hosts through shared environments [12, 115]. For instance, certain risk behaviours (e.g., wallowing, drinking) can promote frequent and prolonged contact with various infection sources and affect infection outcome and excretion patterns per host. This topic needs further research as it remains poorly understood in the Iberian context; third, since indirect transmission depends on *M. bovis* survival time in environments, the use of CTW is crucial for generating reliable estimates. However, as *M. bovis* can survive for extended periods, depending on climate, substrates, and others [116], important questions arise: where should the baseline (CTW, in time axis) be established in a given context? Should the infectious period be based on the average environmental persistence of *M. bovis*? Should we examine the frequency of indirect interactions that occurred within a plausible range of CTW's, according to hosts, to better define baselines? Should different CTW estimates based on *M. bovis* survival be considered across various substrates associated with sampling sites? [34]. Progress in addressing these important questions has been made, with a few studies pioneering the implementation of CTW's through different approaches to define host interactions [31, 63]. Adopting similar frameworks, with environmental survival as a gold-standard metric, will improve integration and comparison of results across studies. Nevertheless, researchers will also benefit from studies exploring multiple CTW's as a function of interaction gradients, as well as the definition of CTW's according to sampling spatial conditions [33]. This is key to developing general theory on this topic, also applicable to other infectious diseases at the wildlife–cattle interface.

## 5. Conclusions

This is the most comprehensive study carried out in Portugal focusing on species indirect interactions in an endemic TB context, and identifying the most likely key ecological factors driving these interactions across shared environments. Our study confirmed that the availability of natural food and water was a main driver of wildlife–cattle interactions, while wildlife indirect interactions were more associated with human disturbance. However, other ecological hypotheses influenced indirect interaction patterns, suggesting that the conditions favouring the complex transmission of *M. bovis* are determined by multiple factors, depending on the host species and season. Future studies should combine interaction data with the extent of environmental contamination with *M. bovis* to properly assess transmission risk in multi-host communities. Furthermore, the composition and structure of multi-host communities determining complex interaction patterns in space-time axes should also be considered when establishing priority measures for disease control in shared environments.

## Figures and Tables

**Table 1:** Study hypotheses and description of the eco-environmental predictors used for modelling interspecies interactions.

Hypothesis	Inclusion rationale	Prediction	Predictor acronym	Description
H1. Human disturbance	Wildlife species tend to show a spatial–temporal avoidance of humans and to humans-related activities, which in turn may influence patterns of interspecies interactions [64, 65, 66]	We expect a negative association between human disturbance and abundance of interspecies interactions. We also expect that a greater human presence may also imply a greater presence of domestic species which increases the likelihood of wildlife–cattle interactions	Dist_houses	Distance of camera sites to the nearest artificial houses/facilities (m)
			Dist_hunt	Distance of camera sites to the nearest hunting site (m)
			DensRoad	Density of unpaved roads within 100, 250, and 500 m spatial scales around camera sites
			Human_days	Number of days with occurrence of humans (a proxy for human presence)

H2. Landscape composition	The occurrence and distribution of species depend on their habitat requirements, and thus landscape context may be a key driver for interspecies interactions [6, 56, 65]	We predict that landscape composition is the most important mechanism driving interspecies interactions. We expect a positive relationship between forest and heterogeneous areas and wildlife interactions; and a positive relationship between agro-dominated areas and wildlife–cattle interactions	Agro	Percentage of agroforest land (holm oak stands with low or absent shrub cover due to grazing and other pastoral activities) within 100, 250, and 500 m spatial scales around camera sites (%)
			Forest	Percentage of forest (holm oak stands or mixed woodland patches with high shrub cover) within 100, 250, and 500 m spatial scales around camera sites (%)
			TreeD	Proportion of tree cover density within 100, 250, and 500 m spatial scales around camera sites (%)
			Dist_edgeF	Distance of camera sites to the nearest edge of forest patches (m)
			Shidi	Shannon's landscape diversity index within 100, 250, and 500 m spatial scales around camera sites

H3. Topography	Terrain features are important drivers that regulate species co-occurrence and thus influence shared space among host species [64, 67]	We expect a negative relationship between topography-based predictors and species interactions	Altitude	Terrain altitude within 100, 250, and 500 m spatial scales around camera sites
			Rugg	Terrain ruggedness index within 100, 250, and 500 m spatial scales around camera sites
			Slope	Topographic slope within 100, 250, and 500 m spatial scales around camera sites

H4. Weather	Weather conditions shape species activity and, in turn, can drive interactions among hosts across space and time gradients [24, 60, 68]	We predict that weather conditions exert positive or negative effects on interspecies interactions, being species-specific and season dependent	Temp	Minimum monthly temperature (°C), used in the wet season
			Temp	Maximum monthly temperature (°C), used in the dry season
			Rain	Total monthly accumulated precipitation (mm)

H5. Natural food and water resources	Food and water resources can facilitate species aggregation, thus being an important factor shaping spatial and temporal patterns of interactions between mammal host species [24, 28]	We predict that food-rich areas, along with water abundance, have a positive influence on interspecies interactions, particularly during the dry season	Water_cont	Water content at each camera site (mean monthly water area size; m^2^), calculated by visual estimation in the field
			Station_site	Typology of the camera sites: control sites and water sites
			NDVI	Normalised Difference Vegetation Index within 100, 250, and 500 m spatial scales around camera sites

**Table 2:** Summary of the hypotheses (H) tested and predictors (highlighted in bold) significantly related to wildlife–cattle species pair interactions.

Species pair	Season	H	Model id	Null model AICc	Model ref AICc	Model AICc	DeltaAIC	Predictors	Coeff.	CI: 95%	IRR
BT_CE	Wet	H2	BTCE_wH2	1,110.9	601.6	598.1	3.5	**BT abundance**	0.945	(0.792; 1.098)	2.573
								**CE abundance**	1.021	(0.901; 1.141)	2.776
								**Forest_100**	0.175	(0.023; 0.326)	1.191

BT_CE	Wet	H4	BTCE_wH4	1,110.9	601.6	597.0	4.6	**BT abundance**	0.871	(0.726; 1.015)	2.388
								**CE abundance**	1.065	(0.944; 1.186)	2.902
								**Temp**	−0.088	(−0.155; −0.020)	0.916

BT_CE	Wet	H5	BTCE_wH5	1,110.9	601.6	594.7	6.9	**BT abundance**	0.937	(0.794; 1.080)	2.552
								**CE abundance**	1.090	(0.973; 1.207)	2.974
								**Station_site: water**	−0.361	(−0.595; −0.127)	0.697

BT_CE	Dry	H2	BTCE_dH2	248.7	165.5	161.6	3.9	**BT abundance**	0.626	(0.246; 1.005)	1.870
								**CE abundance**	1.363	(0.949; 1.778)	3.908
								**TreeD_100**	−0.868	(−1.659; −0.077)	0.420

BT_CE	Dry	H4	BTCE_dH4	248.7	165.5	160.9	4.6	**BT abundance**	1.071	(0.561; 1.580)	2.917
								**CE abundance**	1.362	(0.936; 1.788)	3.902
								**Rain**	0.314	(0.061; 0.568)	1.369

BT_CE	Dry	H5	BTCE_dH5	248.7	165.5	151.3	14.2	**BT abundance**	0.855	(0.402; 1.309)	2.352
								**CE abundance**	1.248	(0.852; 1.643)	3.483
								NDVI_500	0.198	(−0.074; 0.471)	1.219
								**Water_cont**	0.878	(0.367; 1.388)	2.405

BT_SS	Wet	H1	BTSS_wH1	1,042.8	629.2	624.6	4.6	**BT abundance**	0.743	(0.611; 0.875)	2.103
								**SS abundance**	1.013	(0.904; 1.123)	2.754
								**Human_days**	−0.180	(−0.321; −0.039)	0.836

BT_SS	Wet	H2	BTSS_wH2	1,042.8	629.2	624.7	4.5	**BT abundance**	0.796	(0.662; 0.929)	2.216
								**SS abundance**	0.998	(0.896; 1.100)	2.713
								**Agro_100**	−0.141	(−0.247; −0.035)	0.868

BT_SS	Wet	H5	BTSS_wH5	1,042.8	629.2	625.7	3.5	**BT abundance**	0.707	(0.580; 0.833)	2.027
								**SS abundance**	1.052	(0.945; 1.159)	2.863
								**NDVI_100**	−0.088	(−0.162; −0.014)	0.915

BT_SS ^*∗*^	Dry	—	—	—	—	—	—	—	—	—	—

BT_VV	Wet	H1	BTVV_wH1	1,386.5	688.3	683.4	4.9	**BT abundance**	0.985	(0.841; 1.129)	2.678
								**VV abundance**	1.013	(0.908; 1.119)	2.755
								**Human_days**	−0.110	(−0.193; −0.026)	0.896

BT_VV	Wet	H5	BTVV_wH5	1,386.5	688.3	681.9	6.4	**BT abundance**	0.983	(0.841; 1.125)	2.671
								**VV abundance**	0.999	(0.897; 1.102)	2.717
								**NDVI_500**	−0.073	(−0.137; −0.008)	0.930
								**Station_site: water**	−0.308	(−0.571; −0.045)	0.735

BT_VV	Dry	H1	BTVV_dH1	287.8	180.3	175.9	4.4	**BT abundance**	0.752	(0.490; 1.013)	2.120
								**VV abundance**	1.551	(1.215; 1.887)	4.715
								**DensRoad_250**	−0.384	(−0.682; −0.086)	0.681

BT_VV	Dry	H4	BTVV_dH4	287.8	180.3	171.8	8.5	**BT abundance**	1.026	(0.672; 1.380)	2.789
								**VV abundance**	1.550	(1.179; 1.921)	4.711
								**Rain**	0.296	(0.111; 0.481)	1.344

BT_VV	Dry	H5	BTVV_dH5	287.8	180.3	173.1	7.2	**BT abundance**	0.966	(0.643; 1.289)	2.627
								**VV abundance**	1.651	(1.262; 2.040)	5.211
								**NDVI_100**	0.360	(0.124; 0.596)	1.433

BT_MM	Wet	H2	BTMM_wH2	651.1	371.3	368.5	2.8	**BT abundance**	0.920	(0.703; 1.137)	2.508
								**MM abundance**	1.174	(1.037; 1.312)	3.236
								**Agro_100**	−0.206	(−0.391; −0.020)	0.814

BT_MM ^*∗*^	Dry	—	—	—	—	—	—	—	—	—	—

For each species pair and season, we provided the best model according to the model's AICc (Akaike's information criterion adjusted for small sample sizes). The AICc of the reference model and the null model are also provided. DeltaAICc (*Δ*AICc) was obtained between the reference model and each best model for a given hypothesis. The coefficients (Coeff.) and corresponding 95% confidence intervals (CI: 95%) for each tested predictor are presented. Incidence rate ratios (IRR) are reported as exponentiated results.  ^*∗*^ (asterisk) was used to mark species pairs and seasons for which we did not find asignificant association with the tested hypotheses.

**Table 3:** Summary of the hypotheses (H) tested and predictors (highlighted in bold) significantly related to wildlife species pair interactions.

Species pair	Season	H	Model identifier	Null model AICc	Model ref AICc	Model AICc	DeltaAIC	Predictors	Coeff.	CI: 95%	IRR
CE_SS	Wet	H1	CESS_wH1	928	539.7	537.8	2.0	**CE abundance**	0.740	(0.632; 0.847)	2.095
								**SS abundance**	0.788	(0.672; 0.903)	2.198
								**Dist_houses**	0.083	(0.001; 0.164)	1.086

CE_SS	Wet	H4	CESS_wH4	928	539.7	537.1	2.6	**CE abundance**	0.747	(0.638; 0.856)	2.111
								**SS abundance**	0.752	(0.635; 0.870)	2.122
								**Temp**	−0.082	(−0.157; −0.007)	0.921

CE_SS ^*∗*^	Dry	—	—	—	—	—	—	—	—	—	—

CE_VV	Wet	H1	CEVV_wH1	906.1	529.9	527.4	2.5	**CE abundance**	0.933	(0.832; 1.033)	2.542
								**VV abundance**	0.892	(0.784; 1.000)	2.440
								**DensRoad_100**	−0.116	(−0.224; −0.008)	0.891

CE_VV ^*∗*^	Dry	—	—	—	—	—	—	—	—	—	—

CE_MM ^*∗*^	Wet	—	—	—	—	—	—	—	—	—	—

CE_MM	Dry	H4	CEMM_dH4	205.4	90.4	83.7	6.7	**CE abundance**	0.757	(0.295; 1.218)	2.131
								**MM abundance**	1.043	(0.736; 1.350)	2.838
								**Rain**	0.455	(0.118; 0.791)	1.575

VV_SS	Wet	H1	VVSS_wH1	859.4	546.1	543.8	2.3	**VV abundance**	0.837	(0.708; 0.966)	2.310
								**SS abundance**	0.920	(0.788; 1.051)	2.509
								**Human_days**	−0.161	(−0.318; −0.004)	0.851

VV_SS	Wet	H2	VVSS_wH2	859.4	546.1	538.7	7.4	**VV abundance**	0.855	(0.737; 0.972)	2.350
								**SS abundance**	0.929	(0.807; 1.051)	2.532
								Agro_100	−0.091	(−0.191; 0.008)	0.913
								**Shidi_100**	0.108	(0.024; 0.192)	1.114

VV_SS	Dry	H1	VVSS_dH1	251.2	136.3	132.3	4.0	**VV abundance**	1.220	(0.838; 1.602)	3.387
								**SS abundance**	1.054	(0.762; 1.345)	2.869
								**Dist_houses**	−0.376	(−0.708; −0.043)	0.687

SS_MM ^*∗*^	Wet	—	—	—	—	—	—	—	—	—	—

SS_MM	Dry	H1	SSMM_dH1	173.4	94.3	89.2	5.1	**SS abundance**	1.111	(0.676; 1.545)	3.036
								**MM abundance**	1.065	(0.811; 1.319)	2.900
								**DensRoad_100**	−0.635	(−1.122; −0.147)	0.530

VV_MM ^*∗*^	Wet	—	—	—	—	—	—	—	—	—	—

VV_MM ^*∗*^	Dry	—	—	—	—	—	—	—	—	—	—

For each species pair and season, we provided the best model according to the model's AICc (Akaike's information criterion adjusted for small sample sizes). The AICc of the reference model and the null model are also provided. DeltaAICc (*Δ*AICc) was obtained between the reference model and each best model for a given hypothesis. The coefficients (Coeff.) and corresponding 95% confidence intervals (CI: 95%) for each tested predictor are presented. Incidence rate ratios (IRR) are reported as exponentiated results. ^*∗*^ (asterisk) was used to mark species pairs and seasons for which we did not find a significant association with the tested hypotheses.

## Data Availability

The data associated with this research are available from the corresponding author upon reasonable request.

## Supplementary Materials

Supplementary Materials Additional supplementary information includes temperature/rainfall comparisons between Ciudad real (Spain) and Barrancos (Portugal), and DHARMa diagnostic plots of the tested models.
