# Supplementary material for: Host-, Environment-, or Human-Related Effects Drive Interspecies Interactions in an Animal Tuberculosis Multi-Host Community Depending on the Host and Season
Source: Transbound Emerg Dis. 2024 Jun 10;2024:9779569. doi: 10.1155/2024/9779569 (PMC12017344; doi:10.1155/2024/9779569)
Supplement: Supplementary Materials — Additional supplementary information includes temperature/rainfall comparisons between Ciudad real (Spain) and Barrancos (Portugal), and DHARMa diagnostic plots of the tested models. [file 9779569.f1.docx]

**Supplementary Material to “Host, environment, or human-related effects drive interspecies interactions in an animal tuberculosis multi-host community depending on the host and season”**

Eduardo M. Ferreira, Mónica V. Cunha, Elsa L. Duarte, Renata Gonçalves, Tiago Pinto, António Mira, Sara M. Santos

Corresponding author: Eduardo M. Ferreira

MED - Mediterranean Institute for Agriculture, Environment and Development & CHANGE – Global Change and Sustainability Institute, University of Évora, Mitra, 7006-554 Évora, Portugal [ferreiraeduardo.mr@gmail.com](mailto:ferreiraeduardo.mr@gmail.com)

This material includes:

**Figure S1**. Comparison of monthly average maximum (T_max) and minimum (T_min) temperatures between Ciudad real (Spain; black and grey lines, respectively) and Barrancos (Portugal; red and blue lines, respectively). Sources: <https://nomadseason.com/climate/spain/castille-la-mancha/ciudad-real.html>, <https://nomadseason.com/climate/portugal/beja/barranco>.

**Figure S2**. Comparison of monthly average rainfall between Ciudad real (Spain; Prec_CR; grey line) and Barrancos (Portugal; Prec_BC; orange line). Sources: <https://nomadseason.com/climate/spain/castille-la-mancha/ciudad-real.html>, <https://nomadseason.com/climate/portugal/beja/barranco>.

**Figure S3**. DHARMa diagnostic plots for the BTCE_wH5 model (species pair: BT_CE; season: wet, hypothesis: H5) showing residual, dispersion, outliers, and zero-inflation fits.

**Figure S4**. DHARMa diagnostic plots for the BTVV_dH2 model (species pair: BT_VV; season: dry, hypothesis: H2) showing residual, dispersion, outliers, and zero-inflation fits.

**Figure S5**. DHARMa diagnostic plots for the VVSS_dH1 model (species pair: VV_SS; season: dry, hypothesis: H1) showing residual, dispersion, outliers, and zero-inflation fits.

**Figure S6.** DHARMa diagnostic plots for the VVSS_wH2 model (species pair: VV_SS; season: wet, hypothesis: H2) showing residual, dispersion, outliers, and zero-inflation fits.


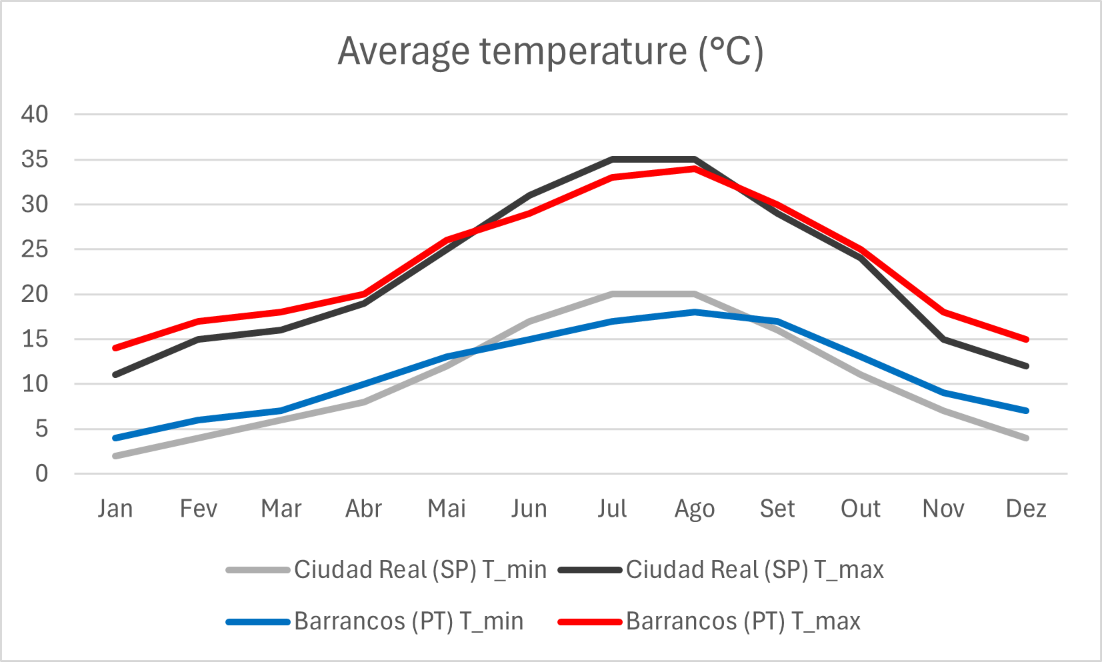


**Figure S1**. Comparison of monthly average maximum (T_max) and minimum (T_min) temperatures between Ciudad real (Spain; black and grey lines, respectively) and Barrancos (Portugal; red and blue lines, respectively). Sources: <https://nomadseason.com/climate/spain/castille-la-mancha/ciudad-real.html>, <https://nomadseason.com/climate/portugal/beja/barranco>.


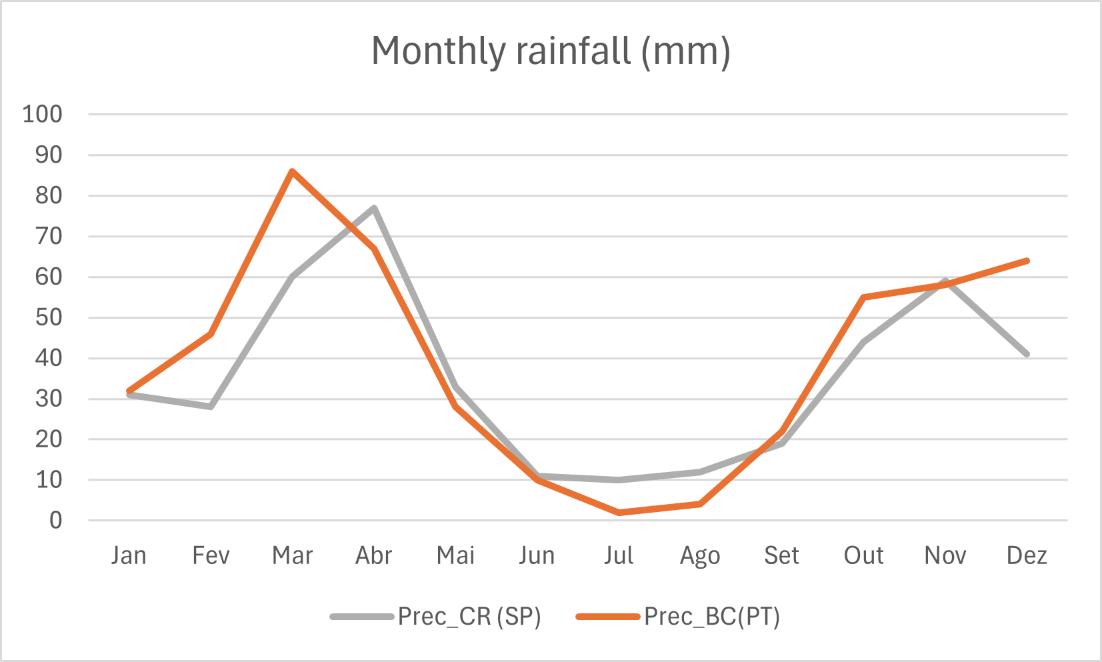


**Figure S2**. Comparison of monthly average rainfall between Ciudad real (Spain; Prec_CR; grey line) and Barrancos (Portugal; Prec_BC; orange line). Sources: <https://nomadseason.com/climate/spain/castille-la-mancha/ciudad-real.html>, <https://nomadseason.com/climate/portugal/beja/barranco>.

| 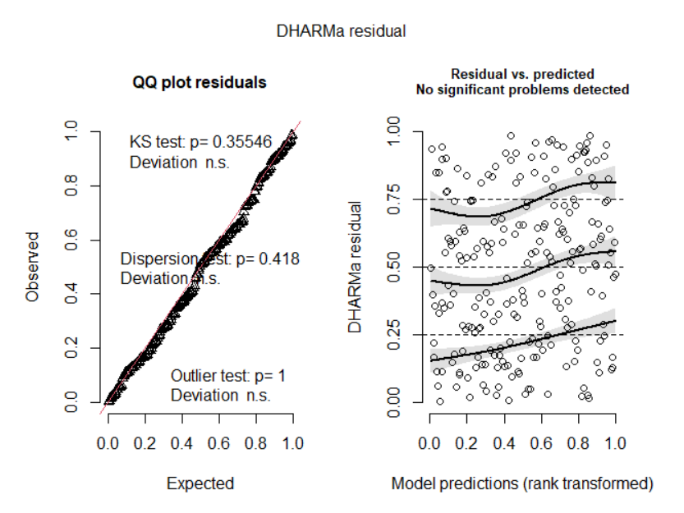 | 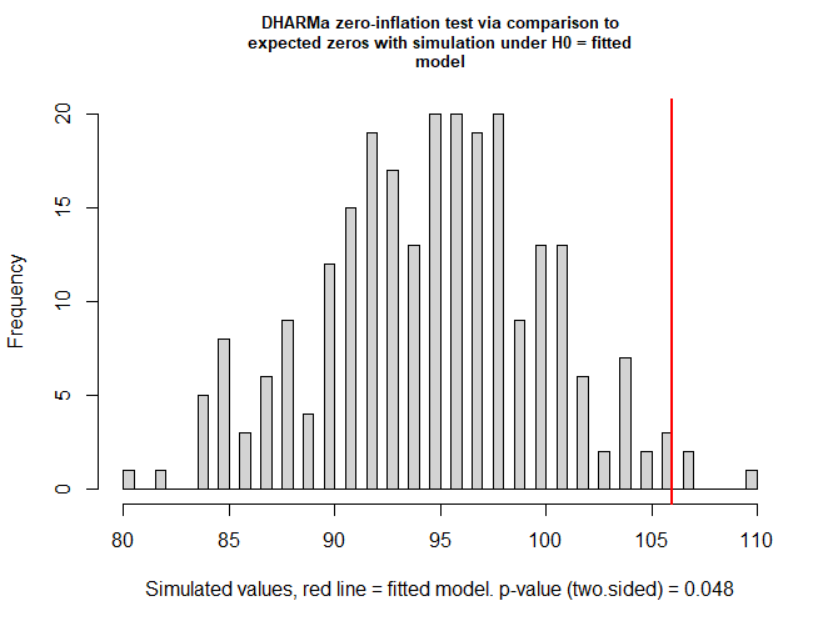 |
| --- | --- |

**Figure S3**. DHARMa diagnostic plots for the BTCE_wH5 model (species pair: BT_CE; season: wet, hypothesis: H5) showing residual, dispersion, outliers, and zero-inflation fits.

| 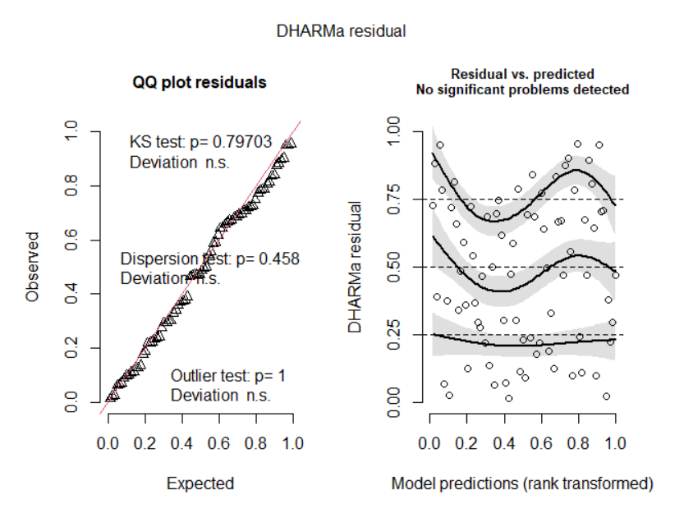 | 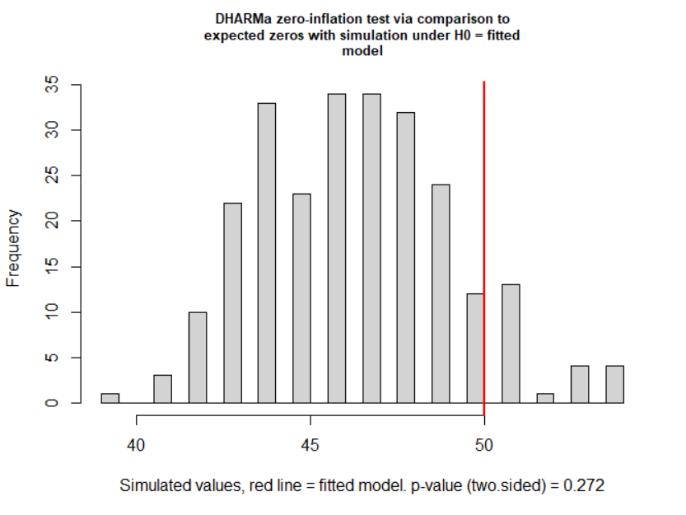 |
| --- | --- |

**Figure S4**. DHARMa diagnostic plots for the BTVV_dH2 model (species pair: BT_VV; season: dry, hypothesis: H2) showing residual, dispersion, outliers, and zero-inflation fits.

| 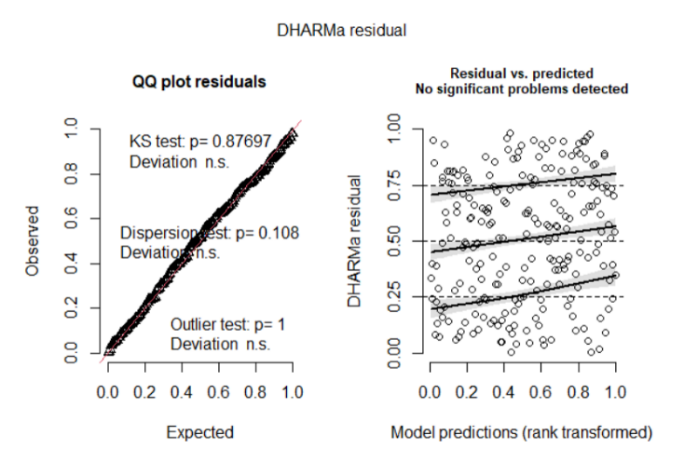 | 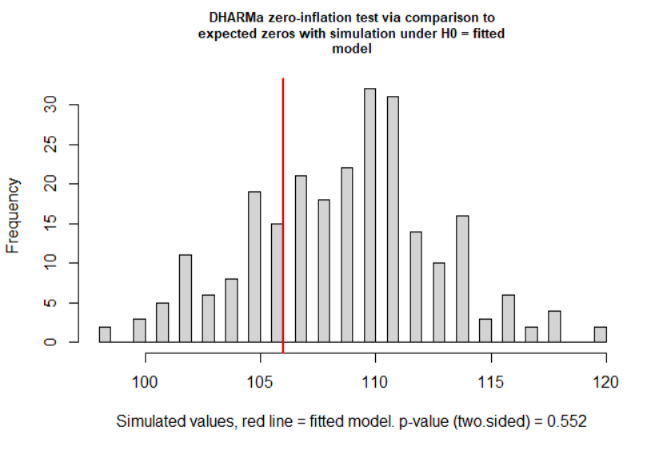 |
| --- | --- |

**Figure S5**. DHARMa diagnostic plots for the VVSS_dH1 model (species pair: VV_SS; season: dry, hypothesis: H1) showing residual, dispersion, outliers, and zero-inflation fits.

| 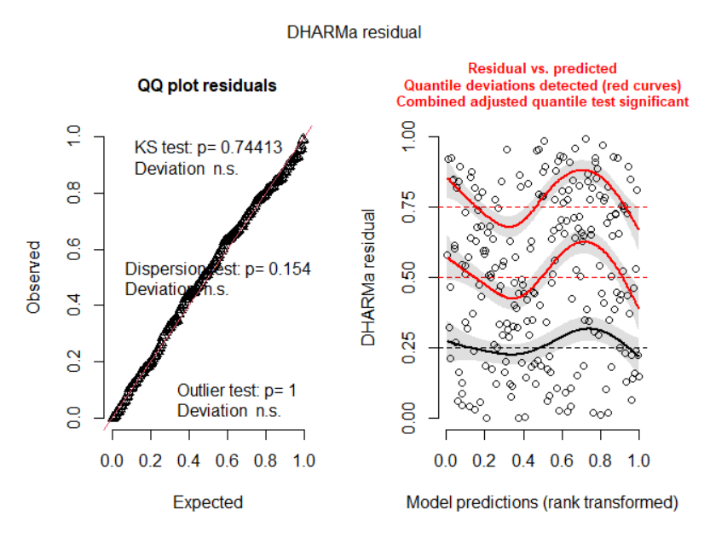 | 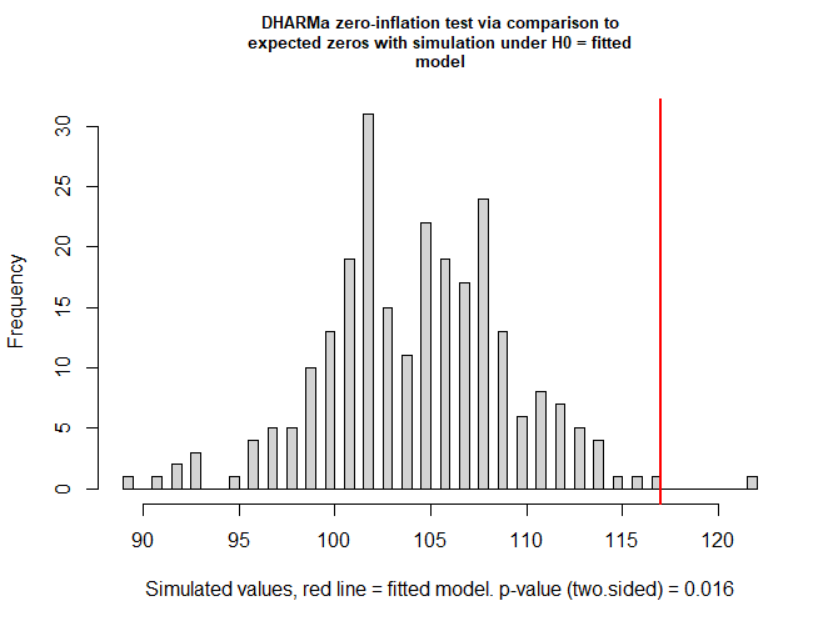 |
| --- | --- |

**Figure S6**. DHARMa diagnostic plots for the VVSS_wH2 model (species pair: VV_SS; season: wet, hypothesis: H2) showing residual, dispersion, outliers, and zero-inflation fits. The examples above not only illustrate the diverse range of patterns observed across all tested models, but also represent the most representative models, considering both studied animal groups (wildlife-cattle and wildlife), seasons and tested hypotheses. In approximately 35% and 20 % of the models generated for wildlife-cattle and wildlife groups, respectively, we identified zero-inflation, indicating a poor fit (see example in Figure.S6). These models were then compared to models incorporating a zero-inflation formula correction [ZIP] (using the argument = ~ animal abundance A and/or animal abundance B^1,2^. While the corrected models demonstrated significant improvements in terms of AICc and no violation of model assumptions, they exhibited instability in the zero-inflation component, with high standard errors associated with the ziformula-variables (likely due to the high complexity of the model structure). Considering that the trends and significance of the predictors (conditional part) remained consistent with or without the correction ZIP formula, we retained the uncorrected and simpler models for further analyses.^1^ Brooks ME, Kristensen K, van Benthem KJ, Magnusson A, Berg CW, Nielsen A, Skaug HJ, Mächler M, Bolker BM (2017) glmmTMB balances speed and flexibility among packages for zero-inflated generalized linear mixed modeling. R Journal 9: 378–400. ^2^ Santon M, Korner-Nievergelt F, Michiels NK, Anthes N (2023) A versatile workflow for linear modelling in R. Frontiers in Ecology and Evolution 11: 1065273.
